# The Absence of the Arabidopsis Chaperone Complex CAF-1 Produces Mitotic Chromosome Abnormalities and Changes in the Expression Profiles of Genes Involved in DNA Repair

**DOI:** 10.3389/fpls.2017.00525

**Published:** 2017-04-11

**Authors:** Javier Varas, Juan L. Santos, Mónica Pradillo

**Affiliations:** Departamento de Genética, Facultad de Biología, Universidad Complutense de MadridMadrid, Spain

**Keywords:** *Arabidopsis thaliana*, DNA double-strand breaks, DNA repair, homologous recombination, non-homologous end-joining

## Abstract

Chromatin Assembly Factor 1 (CAF-1) is an evolutionary conserved heterotrimeric chaperone complex that facilitates the incorporation of histones H3 and H4 onto newly synthesized DNA. We demonstrate here that the mutant deficient for the large subunit of the complex, *fas1-4*, and in minor extent, the mutant deficient for the middle subunit, *fas2-1*, display chromosome abnormalities throughout Arabidopsis mitosis. Among them, we observed multicentromeric chromosomes at metaphase, and chromatid bridges and acentric fragments at anaphase-telophase. 45S rDNA and telomeric sequences were frequently involved in bridges and fragments. Gene expression analysis by real-time qPCR has revealed that several genes related to homologous recombination (HR) and alternative non-homologous end-joining (aNHEJ) are overexpressed in *fas1-4*. These results concur with previous studies which have indicated that HR may be involved in the progressive loss of 45S rDNA and telomeres displayed by *fas* mutants. However, increased expression of *PARP1, PARP2*, and *LIG6* in *fas1-4*, and the phenotype shown by the double mutant *fas1 rad51* suggest that aNHEJ should also be responsible for the chromosomal aberrations observed. The activity of different DNA repair pathways in absence of CAF-1 is discussed.

## Introduction

Histone chaperones are key regulators that participate in distinct steps of nucleosome assembly and histone exchange. They display essential roles during DNA replication and DNA repair (Eitoku et al., 2008; Das et al., 2010; Ransom et al., 2010; Burgess and Zhang, 2013). The Chromatin Assembly Factor 1 (CAF-1) is a heterotrimeric chaperone complex which facilitates the association and deposition of (H3-H4)_2_ histone tetramers onto newly synthesized DNA (Kaufman et al., 1995; Gaillard et al., 1996). Although CAF-1 is highly conserved, the consequences of its disruption are not identical among different organisms (Kaufman et al., 1997; Nabatiyan and Krude, 2004; Song et al., 2007). The complex is essential during the development in mouse and *Drosophila*, but yeast cells and plants with mutations affecting this complex are viable (Kaufman et al., 1997; Exner et al., 2006; Houlard et al., 2006; Song et al., 2007).

In *Arabidopsis thaliana*, *FASCIATA 1* (*FAS1*) encodes the large subunit of CAF-1. It interacts directly with histone tetramers, and also with the DNA polymerase processivity factor namely *PROLIFERATING CELL NUCLEAR ANTIGEN* (PCNA). *FASCIATA 2* (*FAS2*) and *MULTICOPY SUPRESSOR OF IRA* (*MSI1*) encode the other two subunits of the complex (Ramirez-Parra and Gutierrez, 2007a). *fasciata* mutants exhibit pleiotropic phenotypes that include fasciated stems, defective shoot apical meristems, disrupted leaf phyllotaxy, altered trichome differentiation, narrow and dentate leaves, and short roots (Leyser and Furner, 1992; Kaya et al., 2001; Exner et al., 2006). Cytological studies revealed that *fas1* and *fas2* mutants show an open chromatin conformation, as well as reduced heterochromatin content and dispersed pericentromeric DNA (Kirik et al., 2006; Schönrock et al., 2006). The mutants are hypersensitive to genotoxic agents and display an enhanced frequency of homologous recombination (HR) and T-DNA integration. They also present high levels of H2AX phosphorylation, a marker of DNA double-strand breaks (DSBs), and increased *RAD51* expression compared with wild-type (WT) (Takeda et al., 2004; Endo et al., 2006; Kirik et al., 2006; Ono et al., 2006; Schönrock et al., 2006; Ramirez-Parra and Gutierrez, 2007b). Recently, it has been reported that in *fas* mutants there is also a specific and transgenerational loss of 45S rDNA and telomeric sequences (Mozgová et al., 2010; Jaške et al., 2013; Muchová et al., 2015; Pavlištová et al., 2016). The loss of these sequences is produced during the cell division and it does not occur during meiosis (Muchová et al., 2015; Varas et al., 2015). Additionally, CAF-1 deficiency probably leads to the activation of a G2 checkpoint that triggers the endocycle program (Ramirez-Parra and Gutierrez, 2007b). In *fas* mutants cell cycle arrest only occurs during post-meiotic pollen development resulting in formation of one sperm cell (Chen et al., 2008).

*fas1-4* displays 3% of *FAS1* mRNA expression levels and the truncated protein generated is unable to interact with PCNA and other subunits of the CAF-1 complex (Ramirez-Parra and Gutierrez, 2007a). It appears to be the strongest allele described to date since it exhibits the most severe developmental phenotype and ∼96-fold more intrachromosomal HR events than WT (Kaya et al., 2001; Kirik et al., 2006). This HR rate has also consequences in the repair of the DSBs generated during meiosis (Varas et al., 2015). Here, we have mainly focused our attention on the expression profiles of genes involved in different DNA repair pathways and the mitotic consequences derived from the absence of FAS1. Our results highlight that, in addition to HR, the non-homologous end-joining pathway (NHEJ) could be also involved in the enhanced genome instability displayed by *fas1-4.*

## Materials and Methods

### Plant Materials and Growth Conditions

The majority of the mutants analyzed corresponds to T-DNA insertion lines and belong to Columbia accession (Col-0). The exceptions are *rad51-2*, a T-DNA insertion line generated in Wassilewskija (Ws) background (Pradillo et al., 2012), and *fas2-1*, which has been generated by EMS in Landsberg *erecta* (L*er*) background (Endo et al., 2006). The mutant alleles *rad51-3* (SAIL_873_C08) and *dmc1-2* (SAIL_170_F08) were obtained from the Salk Institute Genomic Analysis Laboratory (SiGnAL^1^; Alonso et al., 2003), and provided by the Nottingham Arabidopsis Stock Centre (NASC). Dr. Crisanto Gutiérrez kindly donated *fas1-4* (SAIL_662_D10) and *fas2-1* (Ramirez-Parra and Gutierrez, 2007b) mutant lines. Since there is a progressive transgenerational loss of some sequences in *fas1-4* it is important to point out that the *fas1-4* plants analyzed in this study were obtained from homozygous plants propagated during nine generations by selfing (G9). Plants were cultivated on a soil mixture of vermiculite and commercial soil (3:1) and grown in a green-house under a 16 h light/8 h dark photoperiod, at 18–20°C with 70% humidity. Plants were genotyped by PCR using primers listed in Supplementary Table S1.

### Cytological Analysis

Fixation of flower buds, spreading preparations of somatic cells, and fluorescence *in situ* hybridization (FISH) were carried out according to Sánchez-Morán et al. (2001). The DNA probes used were: 45S rDNA (pTa71; Gerlach and Bedbrook, 1979), 5S rDNA (pCT4.2; Campell et al., 1992), centromeres (pAL1; Martínez-Zapater et al., 1986), and telomeres (pLT11; Richards and Ausubel, 1988). An Olympus BX-60 microscope equipped with an Olympus DP71 digital camera was used for cytological analysis. Images were processed with Adobe Photoshop CS5.

### RNA Extraction and Real-Time Quantitative PCR

Total RNA was isolated from 10-day-old *fas1-4* seedlings by RNeasy kit (Qiagen). Quantitative PCR was performed with the FastStart TaqMan Probe Master using Universal Probe Library (UPL) probes and specific primers designed by the UPL Assay Design Center^2^. Details about the primers and UPL probes are given in Supplementary Table S2. Relative quantification of mRNA was calculated over a calibrator, after normalization to *ACTIN 2* by the standard curve method (Larionov et al., 2005). Three experimental replicates were carried out for each target gene.

### DNA Damage Sensitivity Assays

Cisplatin [*cis*-diamminedichloroplatinum (II), CDDP] is a chemotherapy drug that causes intra-strands crosslinks in the DNA (Eastman, 1985). During DNA replication, some of these links produce DSBs, which are preferentially repaired by HR (de Silva et al., 2002; Sasaki et al., 2004). To test the sensitivity to CDDP, surface-sterilized *fas1-4* and Col-0 seeds were maintained in sterile water at 4°C for approximately 24 h, and then were sown on plates containing MS agar medium with different CDDP concentrations (0, 30, and 50 μM; Sigma). Values corresponding to number of leaves and percentage of germination were evaluated 14 days after sowing.

Most of the oxidative damage caused by hydrogen peroxide, H_2_O_2_, occurs around the sugar-phosphate backbone in the DNA structure and base excision repair (BER) is the major pathway involved in the repair of this damage (Slupphaug et al., 2003). To check the sensitivity to oxidative damage_,_ 10-day-old *fas1-4* seedlings were immersed in MS liquid medium with increasing concentrations of H_2_O_2_ (0, 2.5, 5, 10, and 20 mM). The fresh weight of the plants was quantified 6 days after treatment.

### Statistical Analyses

Statistical analyses were managed with the software SPSS Statistics 17.0. Qualitative variables are shown as percentages and quantitative variables as mean ± standard error. Chi-squared and Student’s *t*-test were used to compare qualitative and quantitative variables, respectively. To evaluate the relative gene expression differences between mutant and WT plants, 95% confidence intervals were defined for the average expression of each gene.

## Results

### Seed Germination and Flowering Are Delayed in *fas1-4*

Differences in developmental transitions between *fas1-4* and WT plants were exemplified by analyzing seed germination and flowering (**Table 1**). Differences in germination were evident 4 days after sowing, and at the ninth day there were about 20% of non-germinated *fas1-4* seeds (*n* = 300). About 80% of WT plants showed the first inflorescences 31 days after sowing, whereas only 42% of mutant plants present them at this time. Since seed germination and flowering are two critical processes coordinately regulated by genetic and environmental factors, we wonder whether the delay showed by *fas1-4* in the parameters mentioned was related to alterations during the mitotic division.

**Table 1:** Seed germination and flowering time comparisons between *fas1-4* and WT plants.

Days after sowing	WT	*fas1-4*	Sig.
**Days before germination**			
1	0.00 ± 0.00	0.00 ± 0.00	NS
2	0.00 ± 0.00	0.33 ± 0.05	NS
3	0.66 ± 0.05	4.00 ± 0.26	NS
4	48.33 ± 0.25	15.66 ± 0.35	^∗∗∗^
5	92.00 ± 0.00	49.33 ± 1.25	^∗^
6	97.66 ± 0.50	69.33 ± 1.19	^∗^
7	98.00 ± 0.50	72.66 ± 0.70	^∗∗^
8	98.66 ± 0.40	77.00 ± 0.75	^∗^
9	99.33 ± 0.25	80.00 ± 0.75	^∗∗^
**Days before flowering**			
23	0.00 ± 0.00	0.00 ± 0.00	NS
24	0.33 ± 0.33	0.00 ± 0.00	NS
25	2.66 ± 1.20	4.33 ± 1.76	NS
26	9.66 ± 3.17	16.00 ± 5.19	NS
27	34.00 ± 4.04	25.66 ± 7.42	NS
28	58.66 ± 6.22	32.66 ± 7.88	NS
29	67.00 ± 5.77	35.30 ± 5.04	^∗^
30	76.00 ± 5.54	38.40 ± 5.20	^∗∗^
31	79.33 ± 5.36	40.66 ± 5.43	^∗∗^
32	82.00 ± 5.19	46.66 ± 5.49	^∗^


*Mean values and standard errors are depicted. Asterisks indicate *P*-values from Student’s *t*-tests: NS, no significant, ^∗∗∗^*P* < 0.001, ^∗∗^*P* < 0.01, and ^∗^*P* < 0.05.*

### *fas1-4* Displays Chromosomal Aberrations during Mitotic Division

**FIGURE 1:**
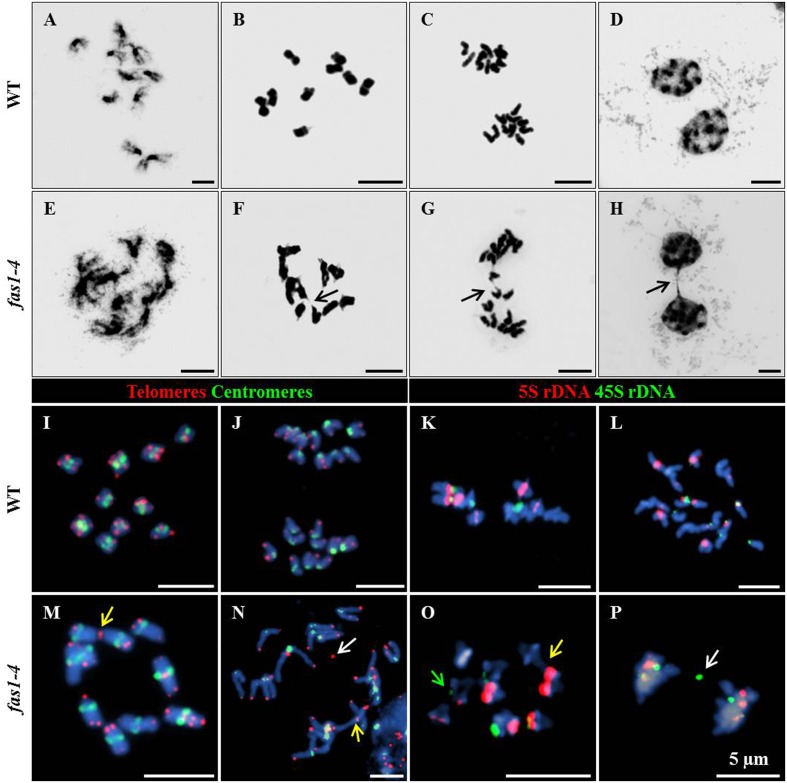
***fas1-4* cells display defects during mitosis.** DAPI-stained chromosome spreads and FISH were performed in somatic cells from young buds of WT **(A–D,I–L)** and *fas1-4* **(E–H,M–P)**. The images show the different mitotic stages: **(A,E)** prometaphase; **(B,F,I,M,K,O)** metaphase; **(C,G,J,L,N,P)** anaphase; **(D,H)** telophase. Arrows in **F–H** point to **(F)** sticky chromosomes, and **(G,H)** chromosome bridges. FISH probes detect **(I,J,M,N)** telomeres (red) and centromeres (green) or **(K,L,O,P)** 5S rDNA (red) and 45S rDNA (green). Yellow arrows denote end-to-end chromosome fusions, white arrows indicate chromosome fragments and the green arrow marks a reduced 45S rDNA region. Scale bars represent 5 μm.

To investigate possible defects during mitosis in *fas1-4*, this division was cytologically analyzed in DAPI-stained chromosome spreads of somatic cells from immature flower buds. This material provides a high metaphase index and is more suitable than root tips in which there are often less than 10 metaphases. In WT plants, mitotic chromosomes are individualized at prometaphase (**Figure 1A**), and their morphology is clearly defined at metaphase (**Figure 1B**). Chromatids segregate to opposite poles at anaphase (**Figure 1C**), and full migration is achieved at telophase (**Figure 1D**). However, altered mitotic stages were observed in *fas1-4* cells. Chromosomes at prometaphase appeared less condensed (**Figure 1E**), 26.31% of the metaphases displayed interchromosomal connections (**Figure 1F**) and chromosome bridges were subsequently observed at anaphase-telophase (**Figures 1G,H** and **Table 2**). To gain insight into these mitotic defects we applied FISH with probes to localize centromeres, telomeres, 45S rDNA, and 5S rDNA sequences. The most relevant results obtained were: (i) there is an intercellular variation in the number of multicentromeric chromosomes at metaphase in *fas1-4*; (ii) chromosome fusions generate mostly dicentric chromosomes; (iii) 45S rDNA and telomeres are frequently involved in the bridges and fragments observed at anaphase (**Figures 1I–P** and Supplementary Figures S1, S2). To determine whether the mitotic phenotype observed in *fas1-4* appears in other mutants deficient for the CAF-1 complex, we also analyzed mitotic cells in *fas2-1* and observed similar abnormalities to those described above (**Table 2** and Supplementary Figure S3). The fact that *fas* mutants suffer ongoing genome destabilization may contribute to the delay observed in the vegetative growth.

**Table 2:** Analysis of mitotic abnormalities in *fas* mutants.

Genetic background	Metaphase						Anaphase		
		
	*n*	I	%	II	%	III	%	*n*	IV	%
WT	104	0	0.00	0	0.00	0	0.00	100	0	0.00
*fas1-4*	76	10	13.16	8	10.53	2	2.63	102	10	9.80
*fas2-1*	82	11	13.41	9	10.98	2	2.44	104	10	9.62
*fas1-4 dmc1-2*	86	11	12.79	9	10.47	2	2.33	110	10	9.09
*fas1-4 rad51-3*	74	18	24.32	10	13.51	8	10.81	108	22	20.37
*fas1-4 rad51-2*	79	22	27.85	12	15.19	10	12.66	101	26	23.76


*n, total cells analyzed; I, number of cells with at least a dicentric chromosome; II, number of cells with dicentric chromosomes; III, number of cells with tricentric or more fused chromosomes; IV, number of cells with either at least one anaphase bridge or a chromosome fragment.*

In order to characterize the different DNA repair pathways that could be induced in *fas1-4* and to get possible explanations for the mitotic abnormalities mentioned above, we conducted different methodological approaches: expression analyses of genes involved in different DNA repair pathways; genetic analyses by crossing *fas1-4* with mutants defective for the recombinases RAD51 and the meiotic-specific DMC1; and DNA damage sensitivity assays.

### Genes Involved in Different DNA Repair Pathways Are Differentially Expressed in *fas1-4*

**FIGURE 2:**
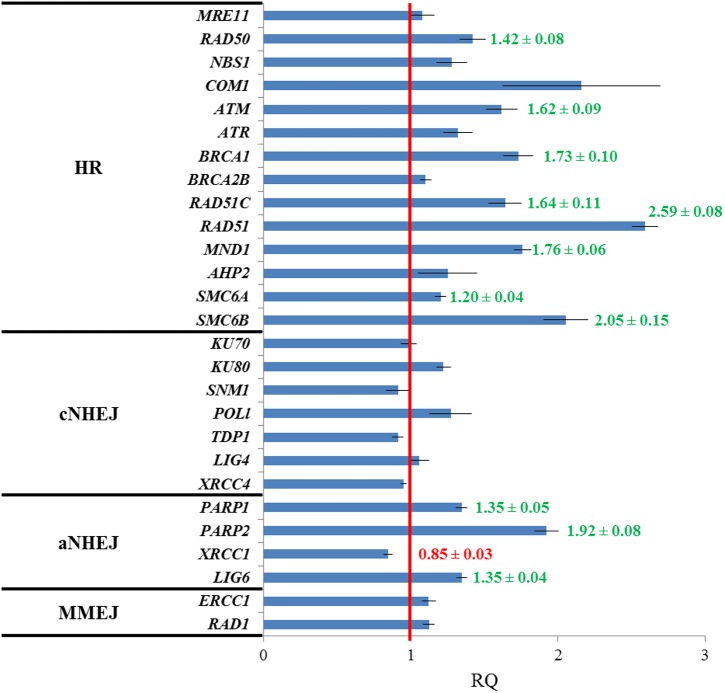
**Genes involved in HR and aNHEJ display different expression profiles in *fas1-4* respect to WT seedlings.** Values are the average of three technical replicates. The red line is the reference for the fold-change respect to the WT after normalization to *ACTIN 2* expression. Genes analyzed have been grouped into four categories: HR, cNHEJ, aNHEJ, and MMEJ. Overexpressed genes are highlighted in green and the underexpressed gene appears in red. Numbers corresponding to fold-changes are only displayed for significant differences. RQ, relative quantity.

Pivotal genes in HR such as *RAD50* (involved in the resection of DSBs), *ATM* (kinase that produces a phosphorylation-mediated signal transduction cascade that leads to the repair of DSBs), *BRCA1* (essential for RAD51 recruitment to sites of DNA damage), *RAD51C* (a *RAD51* paralog), *RAD51* (the main recombinase involved in HR), *MND1* (assists RAD51 in the strand exchange) and *SMC6A* and *SMC6B* (both promote chromatid cohesion after DNA breakage and facilitates HR) were significantly overexpressed in *fas1-4*. Specifically, mRNA levels presented the following fold-changes: 1.42 ± 0.08 (*RAD50*), 1.62 ± 0.09 (*ATM*), 1.73 ± 0.10 (*BRCA1*), 1.64 ± 0.11 (*RAD51C*), 2.59 ± 0.08 (*RAD51*), 1.76 ± 0.06 (*MND1*), 1.20 ± 0.04 (*SMC6A*), and 2.05 ± 0.15 (*SMC6B*). Other genes involved in this DNA repair pathway (*MRE11*, *NBS1, COM1, ATR, BRCA2B*, and *AHP2*) showed high mRNA levels in *fas1-4* seedlings, but they were not statistically significant from those observed in WT seedlings (**Figure 2**).

Neither genes involved in the classical NHEJ (cNHEJ) nor in microhomology-mediated DNA end-joining (MMEJ), a pathway in which DNA repair is achieved using microhomologies localized at both sides of the break (McVey and Lee, 2008; Mladenov and Iliakis, 2011), displayed alterations in their expression. However, we detected significant overexpression of some of the genes involved in an alternative KU-independent pathway, alternative NHEJ (aNHEJ), which does not require the presence of pre-existing microhomologies and may rather rely on LIG1 and LIG6. Main actors in aNHEJ are the poly (ADP-ribose) polymerases *PARP1* and *PARP2* and *XRCC1*, which encodes a protein that acts as scaffold to other DNA repair proteins (Audebert et al., 2008; Charbonnel et al., 2010; Jia et al., 2013; Tallis et al., 2014). We detected statistically higher mRNA levels for *PARP1* (1.35 ± 0.05), *PARP2* (1.92 ± 0.08) and *LIG6* (1.35 ± 0.04), although *XRCC1* was slightly underexpressed (0.85 ± 0.03) (**Figure 2**).

### Mitotic Aberrations in *fas1-4* Are Aggravated in Combination with *rad51* Deficient Alleles

**FIGURE 3:**
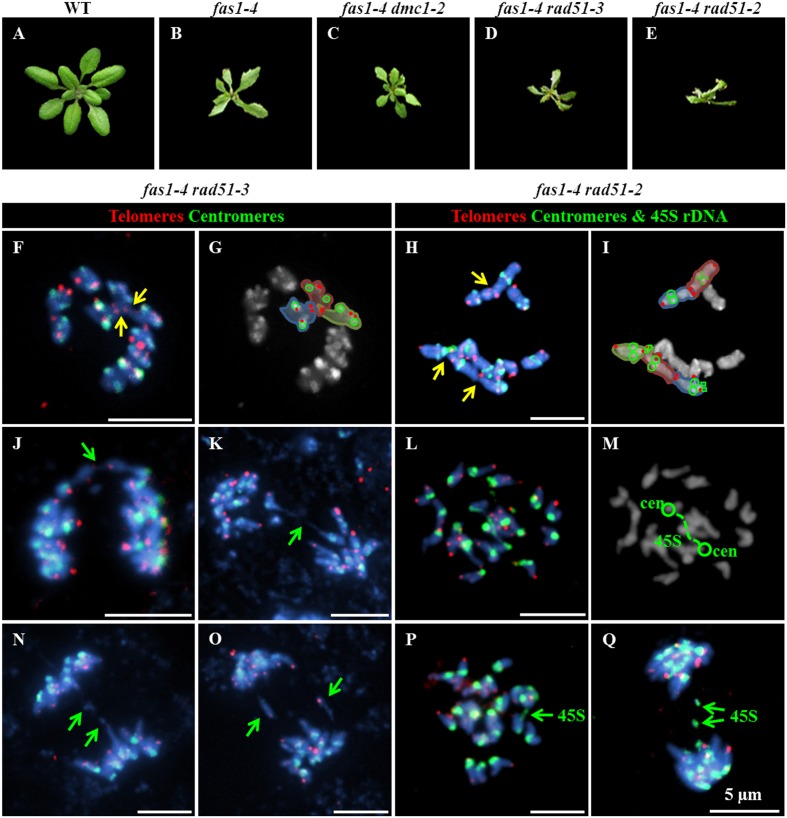
**Absence of RAD51 aggravates the phenotype of *fas1-4*.** Basal rosettes 30 days after sowing in **(A)** WT; **(B)** *fas1-4*; **(C)** *fas1-4 dmc1-2*; **(D)** *fas1-4 rad51-3*; and **(E)** *fas1-4 rad1-2* plants. FISH using telomeric (red), centromeric (green) and 45S rDNA (green) probes in **(F–I)** metaphase and **(J–Q)** anaphase cells from **(F,G,J,K,N,O)** *fas1-4 rad51-3*; and **(H,I,L,M,P,Q)** *fas1-4 rad1-2* plants. Yellow arrows point to end-to-end chromosome fusions; and green arrows denote either chromosome fragments or anaphase bridges. Scale bars represent 5 μm.

To investigate a possible contribution of HR to the mentioned *fas1-4* mitotic defects, three *fas1-4* double mutants were analyzed: *fas1-4 rad51-2* (*rad51-2* is a knockdown allele, KD), *fas1-4 rad51-3* (*rad51-3* is a knockout allele, KO) and *fas1-4 dmc1-2* (*dmc1-2* is a KO allele). RAD51 is an essential protein involved in both mitotic and meiotic DSB repair by HR, whereas DMC1 is a recombinase involved exclusively in meiotic HR. The results obtained confirmed a more drastic phenotype in double mutants involving *rad51* alleles, with smaller leaves and a more pronounced developmental delay respect to the single mutant *fas1-4* (**Figures 3A,E**). The percentage of chromosome aberrations of *fas1-4 dmc1-2* cells at metaphase and anaphase-telophase was similar to that of *fas1-4* (**Table 2** and Supplementary Figure S3, Tables S3–S6). This was an expected result since DMC1 is the meiotic-specific recombinase. However, the percentage of mitotic abnormalities increased significantly in both *fas1-4 rad51-3* (**Figures 3F,G,J,K,N,O** and Supplementary Figure S4) and *fas1-4 rad51-2* respect to *fas1-4* (**Figures 3H,I,L,M,P,Q**, **Table 2**, and Supplementary Figure S5, Tables S3–S6). Surprisingly, the phenotype of the double mutant with the KD allele *rad51-2* was more pronounced than that of the double mutant with the KO allele *rad51-3* (**Figures 3D,E**), which could be attributed to the different genetic backgrounds (the mutant lines *rad51-2* and *rad51-3* belong to Ws and Col-0, respectively). In 20% of the anaphases analyzed in these double mutants, 45S rDNA and telomeric sequences were present in bridges and acentric fragments (**Figures 3O–Q**).

### DNA Damage Sensitivity Assays

**FIGURE 4:**
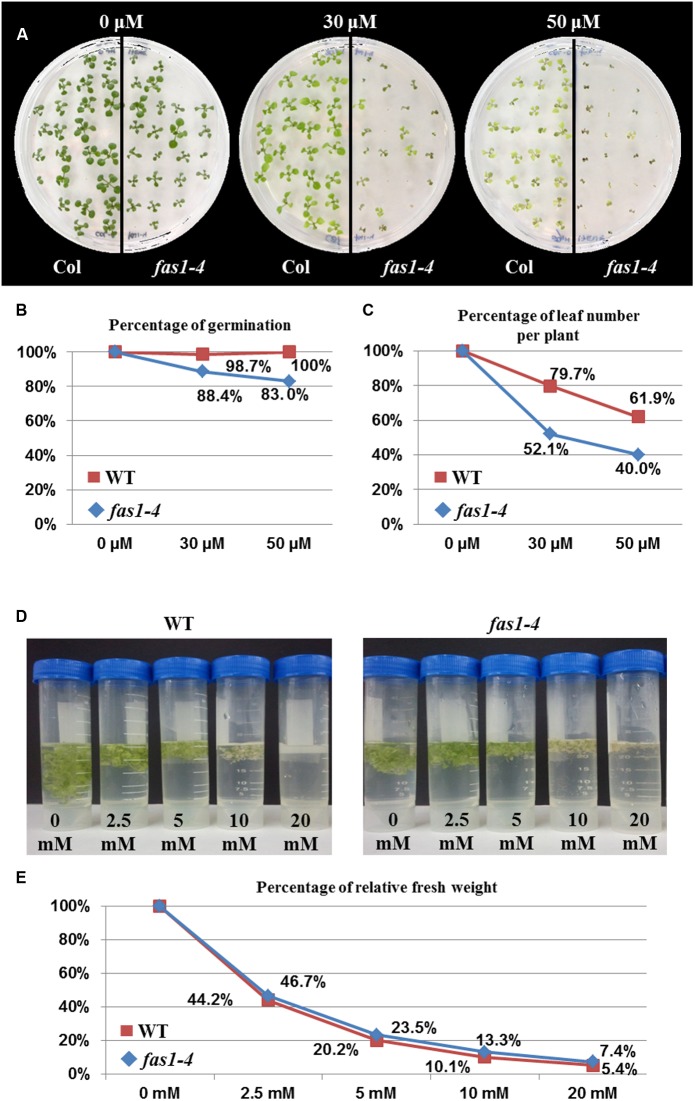
**Absence of FAS1 increases sensitivity to CDDP but not to H_2_O_2_.** **(A)** Phenotypes of 14-day-old seedlings (*fas1-4* and WT) sown on media containing different concentrations of CDDP. **(B)** Percentages of germination. **(C)** Leaf number per plant after treatments with different concentrations of CDDP. The numbers at each dose were scored and put into relation to the leaf numbers of the untreated plantlets of the same line. **(D)** Phenotypes of 16-day-old seedlings (*fas1-4* and WT) 6 days after immersion on liquid media containing different concentrations of H_2_O_2_. **(E)** Percentage of relative fresh weight of the plants 6 days after treatments with different concentrations of H_2_O_2_.

The increase in the percentage of mitotic alterations observed when the HR pathway was non-functional could be explained by the overexpression of some aNHEJ genes. Since *PARP* genes participate in both aNHEJ and BER (Gottlich et al., 1998), we decided to perform two genotoxicity assays to induce either DSBs or single-strand DNA nicks in both *fas1-4* and WT plants. As *fas1-4* showed a slower development and lower germination rate than WT (**Table 1**), we relativized the values of these parameters in relation to those shown by untreated plants. Results obtained revealed that *fas1-4* was more sensitive to CDDP than WT (**Figure 4A**). This difference was higher at enhanced doses for both parameters evaluated: germination rate (**Figure 4B**; 30 μM: $X_{\text{lgl}}^{2}$ = 5.005, *P* = 0.025; 50 μM: $X_{\text{lgl}}^{2}$ = 12.297, *P* < 0.0001) and number of leaves per plant (**Figure 4C**; 30 μM: $X_{\text{lgl}}^{2}$ = 10.793, *P* = 0.001; 50 μM: $X_{\text{lgl}}^{2}$ = 5.623; *P* = 0.017). However, *fas1-4* seedlings were not hypersensitive to H_2_O_2_ (**Figures 4D,E**).

## Discussion

### Somatic Development in *fas1-4* Mutants

The results presented here stress in the generalized somatic developmental delay and growth defects of *fas1-4* mutants and reveal that they could be partially due to mitotic abnormalities (**Figures 1**, **3A,B** and **Table 2**). Additionally, it has been reported that the absence of CAF-1 produces an accumulation of γH2AX histone variant, which marks DSBs (Endo et al., 2006; Amiard et al., 2013). Indeed, *fas* mutants have hypersensitivity to DNase I, γ rays, Zeocin, MMS, UV radiation, mitomycin C and bleomycin (Lowndes and Toh, 2005; Endo et al., 2006; Ono et al., 2006; Ramirez-Parra and Gutierrez, 2007b). Here we add new evidence of enhanced sensitivity of *fas1-4* to cisplatin (**Figure 4A**). This increase in genome instability should be specific to the presence of DSBs and not to single-stranded breaks, since the mutant does not differ from WT in its sensitivity to H_2_O_2_ (**Figures 4D,E**). Consistent with this hypothesis, the absence of the main recombinase involved in HR, RAD51, exacerbates the developmental problems displayed by *fas1-4* (**Figure 3**). Surprisingly, this does not occur when other HR proteins, as RAD51B, are depleted (Muchová et al., 2015).

### Reparation of DSBs Generated as Consequence of Deficient CAF-1 Activity

In eukaryotes, DSBs are mainly repaired by two major pathways: HR and canonical NHEJ. HR predominates in the mid-S and G2 cell cycle phases and requires DNA sequence homology (San Filippo et al., 2008). On the contrary, cNHEJ occurs throughout the cell cycle although is dominant in G0/G1 and G2, and contributes to the repair of DSBs by blunt end ligation independently of sequence homology (Lieber, 2010). Absence of CAF-1 activity leads to genomic instability as consequence of nucleosome assembly failures. In this scenario, chromatin fibers would be more susceptible to mechanical stress and DNA damage (Kaya et al., 2001). Specifically, the increase in somatic HR produced in *fas1-4* would be mainly due to high levels of DSBs derived from stalled DNA replication forks (Gao et al., 2012). Alternatively, it has been proposed that histone loss enhances chromatin dynamics and recombination rates (Hauer et al., 2017).

The importance of HR in the repair of DSBs generated in *fas1-4* is evidenced by two facts. Firstly, by the overexpression of several genes with functions in this pathway (**Figure 2**). Indeed, the highest mRNA levels correspond to *RAD51*, the main gene involved in HR. Secondly, by the increase in the frequencies of mitotic alterations observed in *fas1-4 rad51* (where RAD51 is partially or completely absent) respect to *fas1-4* (**Table 2** and Supplementary Tables S3–S5). In this sense, the RAD51 overexpression generated in *fas1-4* is not enough to solve the genomic instability produced by the absence of the chaperone, since mitotic alterations are observed (**Figure 3**). It is noteworthy the difference in the somatic phenotype between *fas1-4 rad51-2* and *fas1-4 rad51-3* (**Figure 3**). The presence of a small amount of RAD51 protein in *fas1-4 rad51-2* has more drastic consequences that the complete blocking of HR in *fas1-4 rad51-3* (**Figures 4D,E**). This reveals that other DNA pathways (either alternative to HR or negatively regulated by HR) could be contributing to the repair of DNA intermediates generated in *fas1-4*.

Classical non-homologous end-joining, a pathway in which the DSB is repaired without much sequence loss and without microhomologies at the junction, might constitute a potential candidate to explain the presence of multicentromeric chromosomes that will lead to the formation of anaphase bridges. However, genes involved in this pathway such as *KU70*, *KU80* and *LIG4* did not show overexpression respect to WT in *fas1-4*. On the contrary, genes with a role in aNHEJ such as *PARP1, PARP2*, and *LIG6* were overexpressed (**Figure 2**). Through this pathway broken ends are processed until a small number of identical nucleotides are complementary in both strands. The overexpression of *PARP1* was reported previously by Schönrock et al. (2006), but was only related to the activation of a G2 checkpoint. Both *PARP* genes are also potentially involved in BER (Gottlich et al., 1998). However, this pathway seems to be unaffected in *fas1-4* plants (**Figures 4C,D**).

Additionally, Charbonnel et al. (2010) described the existence of a third NHEJ pathway, XRCC1-dependent, named microhomology-mediated end-joining or MMEJ that operates in Arabidopsis. This pathway relies on pre-existing microhomologies around the DSB and is likely to operate through a mechanism related to single strand-annealing (SSA). The same authors have proposed the existence of a hierarchical organization of DSB repair in G2/M nuclei in such a way that cNHEJ acts prior aNHEJ, which can also inhibit MMEJ as seems to occur. In this context, the overexpression of genes involved in aNHEJ together with *XRCC1* underexpression (**Figure 2**) could reflect the disruption of this hierarchical organization of DSB repair in *fas1-4.*

### Deficient CAF-1 Activity and Loss of Repetitive Sequences

In human cells, it has been demonstrated that 45S rDNA repeats but not 5S rDNA repeats are highly sensitive to breaks produced by endonucleases (Warmerdam et al., 2016). NHEJ is the major pathway involved in the repair of these breaks generated in 45S repeat sequences (Harding et al., 2015), although HR is also implicated (van Sluis and McStay, 2015). In these regions, HR could act as an error-prone mechanism if there is an incorrect alignment that originates gain or loss of repeats and structural maintenance chromosome proteins (SMCs) would contribute to this HR-mediated repair (Warmerdam et al., 2016). In this sense, it has been proposed that the progressive loss of 45S rDNA that happens in successive generations in the Arabidopsis *fas1-4* mutant is also HR-mediated, presumably by SSA, since *fas1 rad51b* mutants show a decrease in the rate of rDNA loss (Muchová et al., 2015). However, our results have revealed that *fas1-4 rad51-2* and *fas1-4 rad51-3* present an increase in the number of mitotic abnormalities, with the subsequent loss of rDNA 45S, and more severe developmental defects than the single mutant *fas1-4* (**Figure 3**). These results highlight that other HR-independent DNA repair pathways could also be involved in the progressive loss of rDNA 45S sequences. According to RT qPCR analyses, aNHEJ could be one of these mechanisms (**Figure 2**). Curiously, this pathway is reminiscent of the SSA pathway of HR, since a particular amount of 3′-resection of the broken ends occurs, although the exonucleolytic enzyme complexes implicated are different. Regarding a possible role of SMC proteins in the repair of the breaks generated in the repeats, we have detected overexpression of both *SMC6A* and *SMC6B* in *fas1-4* plants (**Figure 2**). In this context, it has been reported that SMC6A contribute to repair DSBs generated in mutants deficient for NHEJ (Kozak et al., 2008).

In relation to the loss of other repeats, 5S rDNA regions are unaffected in *fas1-4*. However, there is a loss of telomere sequences, although it is produced by a different mechanism from that responsible for the loss of 45S rDNA repeats (Muchová et al., 2015). Jaške et al. (2013), after analyzing single *tert* (telomerase reverse transcriptase) and *fas* mutants, and the corresponding double mutants, concluded that the progressive loss of telomeric DNA along generations in *fas* mutants is partially due to a suboptimal function of telomerase, but they also assumed that a further mechanism that contributes to telomere shortening in *fas* mutants must exist. The existence of telomeric acentric fragments in *fas1-4* anaphases (**Figure 1N**) and the enhanced frequency of these fragments in the double mutant *fas1-4 rad51* (**Figure 3**) suggest the involvement of a RAD51-independent mechanism in telomere shortening.

## Conclusion

Our results suggest that the genome instability produced in *fas1-4* is counteracted by, at least, two different DNA repair pathways: one RAD51-dependent that uses the sister chromatid as template, and another one that is error-prone and dependent on PARP proteins (aNHEJ). This error prone repair pathway could lead to the formation of multicentromeric chromosomes, which are clearly observed at mitotic metaphase in the mutant. Further studies would be required to decipher the specific contribution of these DNA repair pathways, not only in situations in which nucleosome dynamics is affected but also when chromatin conformation is unaffected.

## Author Contributions

JV completed the experiments and performed data analyses. All authors conceived and designed the experiments, and wrote and reviewed the manuscript.

## Conflict of Interest Statement

The authors declare that the research was conducted in the absence of any commercial or financial relationships that could be construed as a potential conflict of interest.

## Footnotes

1 http://signal.salk.edu/cgi-bin/tdnaexpress

2 https://lifescience.roche.com/
