# Supplementary material for: The Absence of the Arabidopsis Chaperone Complex CAF-1 Produces Mitotic Chromosome Abnormalities and Changes in the Expression Profiles of Genes Involved in DNA Repair
Source: Front Plant Sci. 2017 Apr 11;8:525. doi: 10.3389/fpls.2017.00525 (PMC5386969; doi:10.3389/fpls.2017.00525)
Supplement: Supplementary file 1 [file Presentation_1.PDF]

Supplementary Material

The absence of the Arabidopsis chaperone complex CAF-1 produces mitotic chromosome abnormalities and changes in the expression profiles of genes involved in DNA repair

Javier Varas, Juan L. Santos and Mónica Pradillo*

Departamento de Genética, Facultad de Biología, Universidad Complutense, Madrid, Spain

Correspondence:
Dr. Mónica Pradillo
Departamento de Genética
Facultad de Biología
Universidad Complutense de Madrid
C/ José Antonio Novais, 12
Madrid, 28040, Spain
e-mail: pradillo@bio.ucm.es

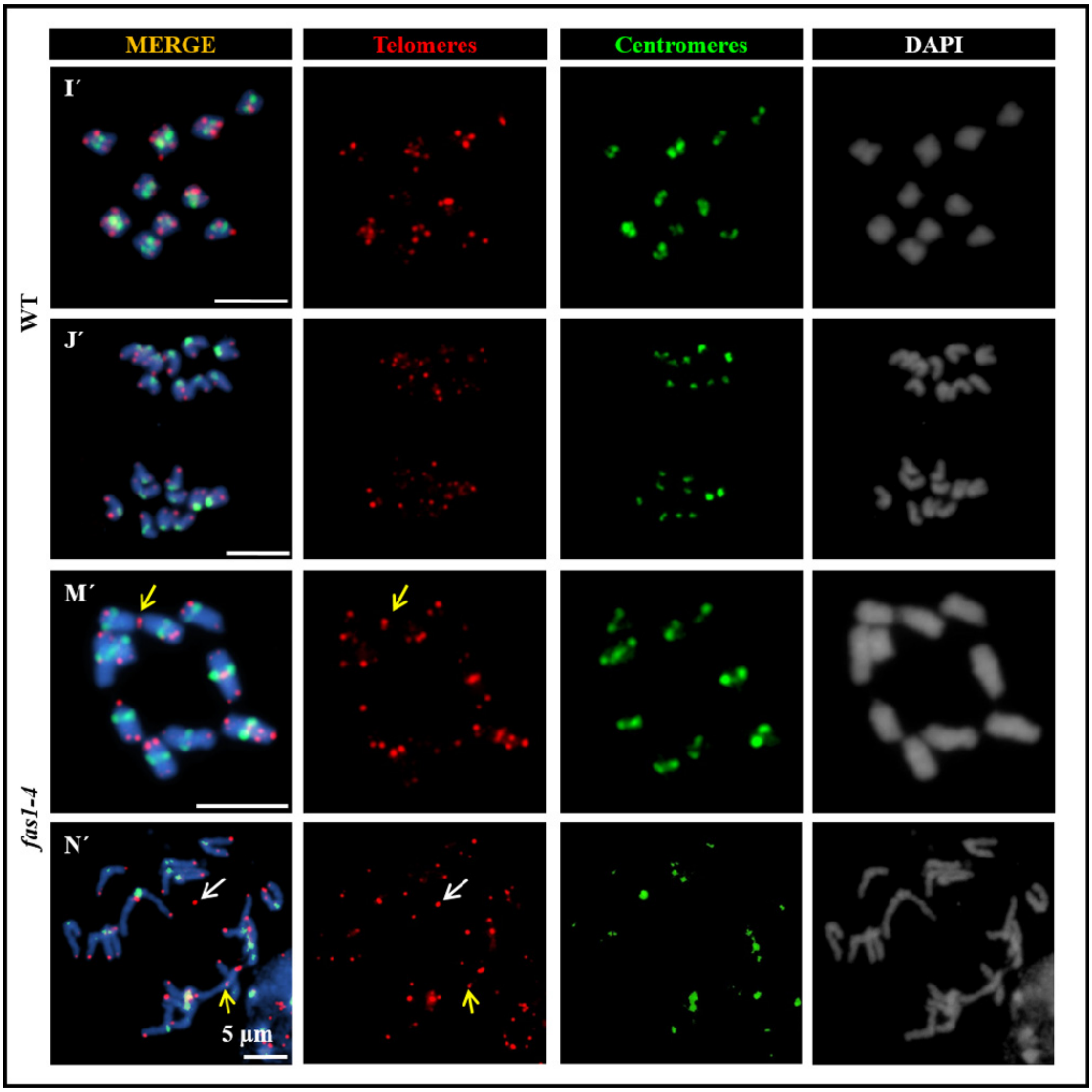

Supplementary Figure S1. Single channel images from WT and *fas1-4* cells after FISH with probes to detect telomeres and centromeres. See Figure 1 for further details. Scale bars represent 5 µm.

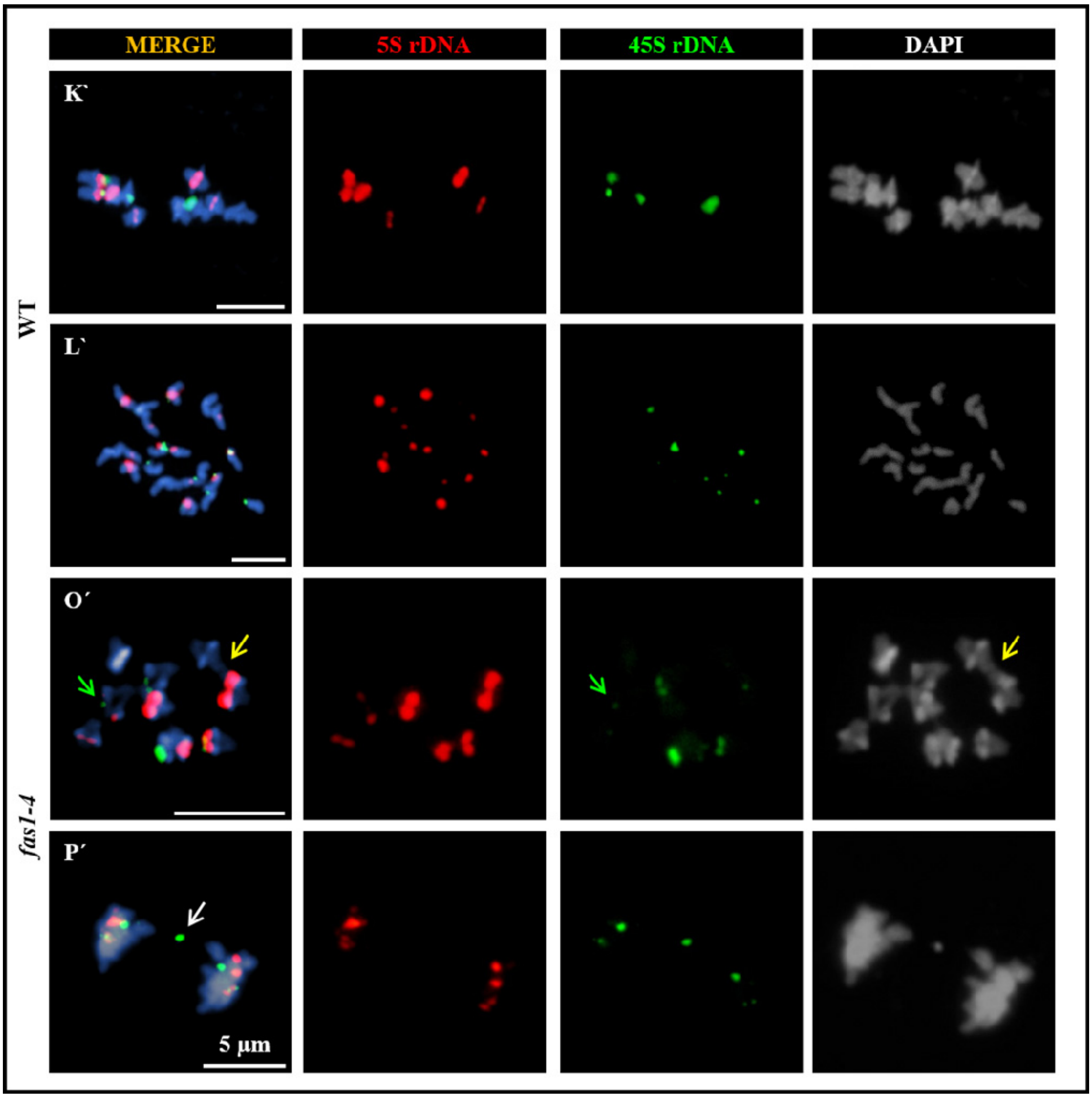

Supplementary Figure S2. Single channel images from WT and *fas1-4* cells after FISH with probes to detect 5S and 45S rDNA regions. See Figure 1 for further details. Scale bars represent 5 µm.

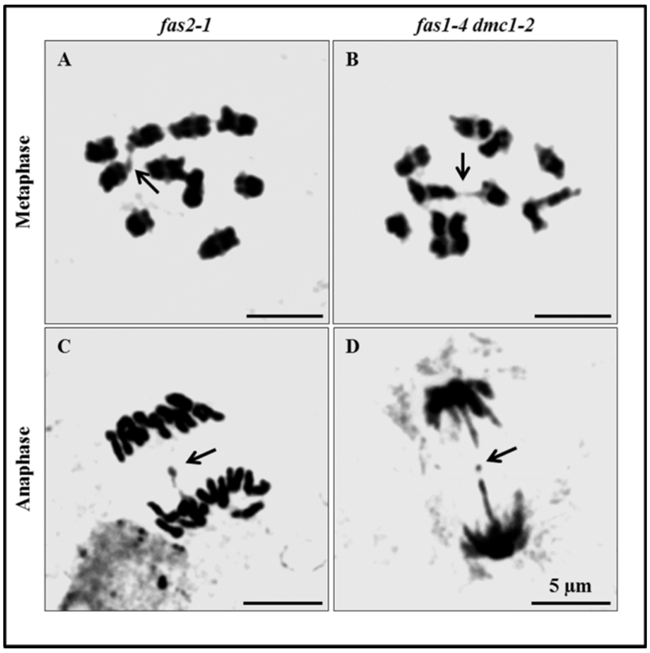

Supplementary Figure S3. Mitosis is *fas2-4* and in the double mutant *fas1-4 dmc1-2*. DAPI stained chromosomes at metaphase and anaphase. Arrows indicate interchromosomal connections and chromosome bridges. Scale bars represent 5 µm.

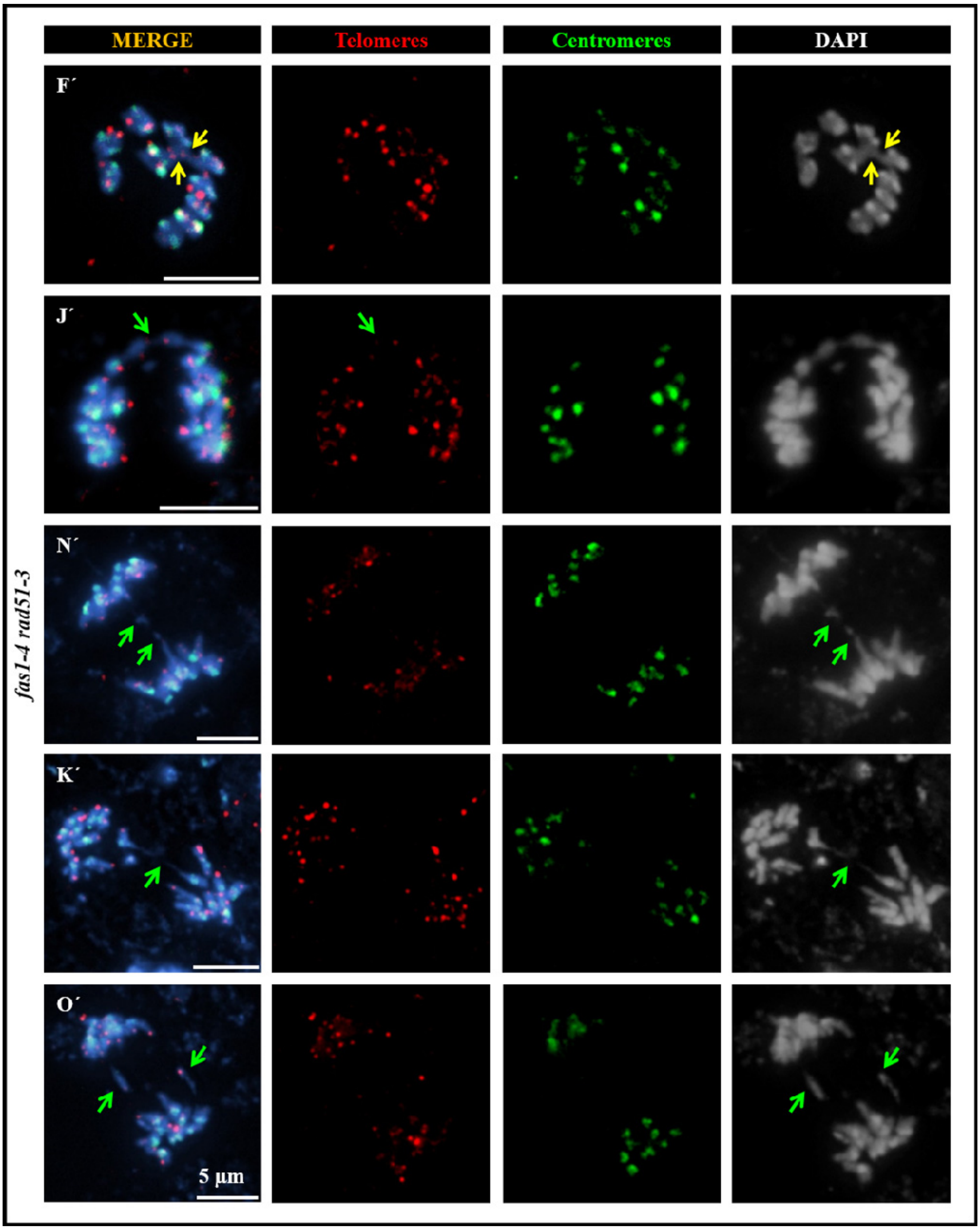

Supplementary Figure S4. Single channel images from *fas1-4 rad51-3* cells after FISH with probes to detect telomeres and centromeres. See Figure 3 for further details. Scale bars represent 5 µm.

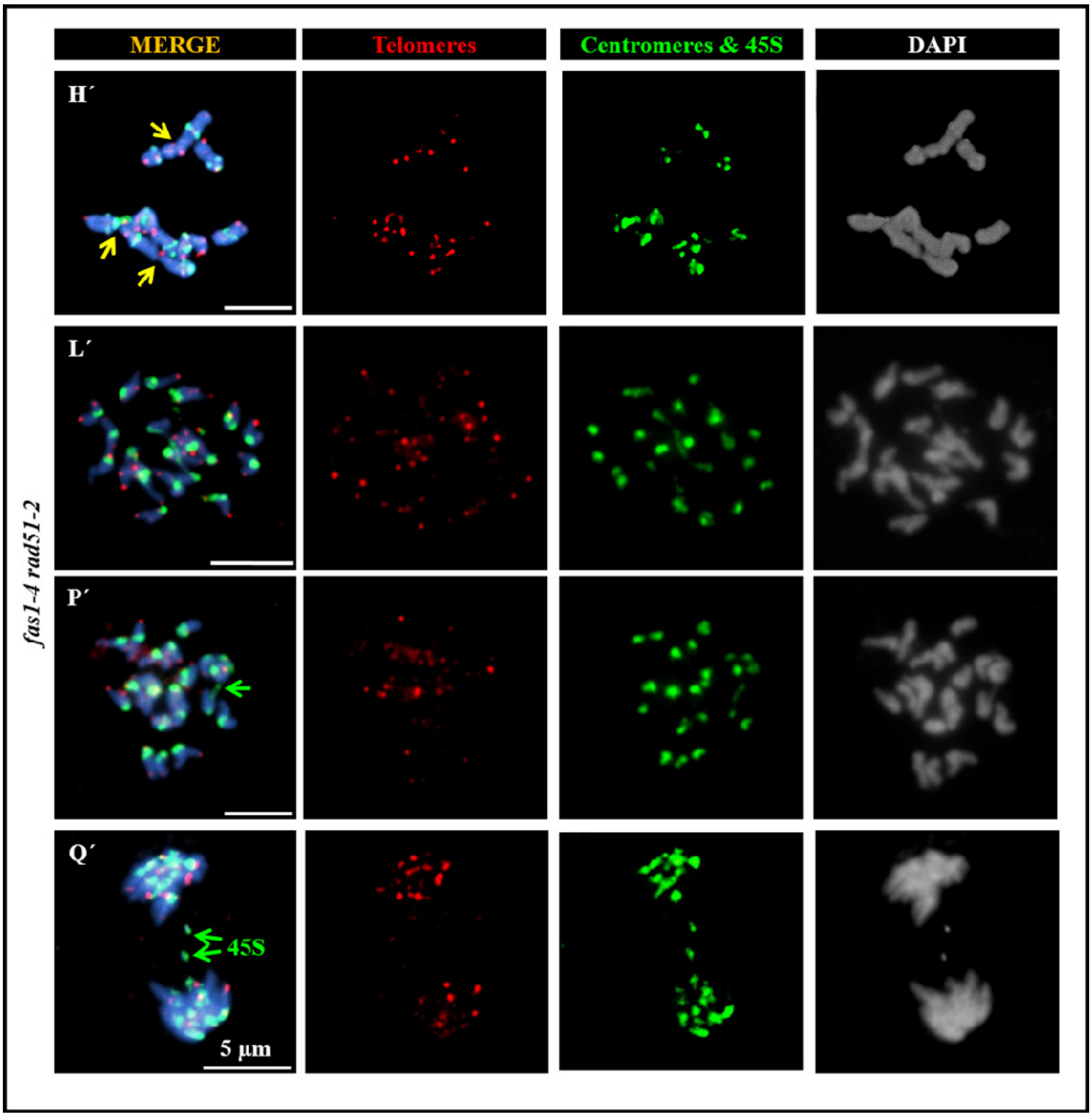

Supplementary Figure S5. Single channel images from *fas1-4 rad51-2* cells after FISH with probes to detect telomeres, centromeres and NORs (45S rDNA). See Figure 3 for further details. Scale bars represent 5 µm.

SUPLEMENTARY TABLES

Supplementary Table S1. Primers used for genotyping.

Mutant	Sequence 5'- 3'	Collection
	Line specific primers	
fas1-4	AAGGAACAAGCCGAGCTAAAG<br>CAAGTTGTAAAGCCACGTCGT	SAIL
rad51-3	TCTCAAGAACTTTGCAAGATGC<br>ATGCCAAGGTTGACAAGATTG	SALK
rad51-2	AAGCATCACCATCTCCAATG<br>GCCCCAGAAAAATCTTCCAG	JP (Spanish collection)
dmc1-2	GACTCATTGTTGCTTGATCCC<br>TCCACTCGGAATAAAGCAATG	SAIL
	T-DNA specific primers	
**LBb1.3**	ATTTTGCCGATTTCGGAAC	SALK
**LB2-SAIL**	GCTTCCTATTATATCTTCCCAAATTACCAATACA	SAIL
**LB2**	TGCCAGGTGCCCACGGAATAG	JP

Supplementary Table S2. Primers and probes used in the RTqPCR to analyze the expression of genes involved in DSB repair.

	Gene	AGI code	Sequence 5´- 3´	UPL probe	Length (bp)
HR	***MRE11***	At5g54260	GTTTCCGCCAGTCTCAAAGA<br>TTCTCCAATGGTGGAAGCA	8	74
	RAD50	At2g31970	GCAGTGCAGGTCAAAAGGTT<br>GGCCCATCCAGGTTTGTAG	136	118
	NBS1	At3g02680	TGCGAAGGATCCATACAAAGA<br>AAGTCCTCTGCAATGGCTTC	38	99
	COM1	At3g52115	CAGCATGAGAAATCAGCAATCT<br>CGAGCCAGTACCAATTCTCAC	68	93
	ATM	At3g48190	AGGGTGGTGAGATGAGAAGC<br>TCTGTGTCAATTGCGTCTTGT	98	67
	ATR	At5g40820	TTCAGCGCCCAAAGAAGA<br>GGCTTGCAGAGGAATGGATA	3	67
	BRCA1	At4g21070	CCAAGAAATTGGTCTTATCTTGC<br>AGTTCCGCAAATTCTGCAAT	100	73
	BRCA2B	At5g01630	CACCTTAAAACCCGCAGTG<br>AGGTGATTTACAAGCACCGATT	140	117
	RAD51C	At2g45280	TCAACTAGCGCTTGCTTTAGG<br>AATACAGAATGACTCGGTTGGTG	54	65
	RAD51	At5g20850	CATGCCACCACAACAAGG<br>ACATGGCGAGCTTATCACTTTAC	91	78
	MND1	At4g29170	TGCGAAAGACAAGATTGGAA<br>AAGTTTCTGGCGAACACTCC	33	88
	AHP2	At1g13330	GAACATTACGGTTGCGTTGTT<br>GGCTGCGTCAACCAATAGTC	53	60
	SMC6A	At5g07660	TGCCTCAAGATGCAACAAAC<br>AAAGTCGAGAAAGACCGTTCC	150	76
	SMC6B	At5g61460	TCGCACGAGAGGATAAAGAAA<br>TGACTCAAAGCCGAGGATG	68	106
cNHEJ	***KU70***	At1G16970	TTGCAAAAACCGGAAAGC<br>ACGAGGAGATTGTTGGCAGT	114	76
	KU80	At1g48050	GGAATGTGTACTCGCTCTTCG<br>GGCATAACTTGAATAGATGGTTCA	125	92
	SNM1	At3g26680	CGGAGATTTCAGGGCTTCTA<br>AACACATCTTCTTTAGAAGGGAATTT	55	129
	POLλ	At1g10520	CGTGTTGAACAGAAGGCTGA<br>CAGACTTGCAGTTCCTCTAGCA	33	124
	TDP1	At5g15170	AGACCGAGATTTGCCTGAGA<br>GTAGCCTCGGTCCCATGAC	154	101
	LIG4	At5g57160	TCGCACCTTGTAGTTCTTGC<br>ACGTTTCTCCATTTCGCTGA	29	84
	XRCC4	At3g23100	AGTTTGAGAGTGCAACTTATGCAA<br>TCCTCCTCAACTACTCTCACTGAA	155	109
aNHEJ	***PARP1***	At2g31320	TCAAAGGTCAAGGCTTCTGAG<br>CCTTACTTTCAACAAGAACTGCAA	26	90
	PARP2	At4g02390	GTTCCACTTGGCAAACCAG<br>CGCATCTTGATTTGTTCCAC	125	95
	XRCC1	At1g80420	GGCTTGAGAGTCAGGAGGAA<br>ACTGATCCAATGTCCTGCTTC	12	124
	LIG6	At1g66730	TTGTGAAGGAATCATGGTCAAG<br>TCGCCCAATCCATCTACATAA	43	111
MMEJ	***RAD1***	At5g41150	TCACAAACAATCCACAGAAGGTA<br>CATACTGCGGAGCTCAATTTT	62	77
	ERCC1	At3g05210	GATTGGCGAACGTAAGGTG<br>ACATCCTTCTCAACTGGAGCTT	119	123

AGI, Arabidopsis Genome Initiative; UPL, Universal Probe Library; HR, homologous recombination; cNHEJ, classical-NHEJ; aNHEJ, alternative-NHEJ; MMEJ, microhomology end-joining.

Supplementary Table S3. Statistical comparisons of the number of cells with metaphase aberrations between the mutants analyzed.

Genetic background	WT	*fas1-4*	*fas2-4*	*fas1-4 dmc1-2*	*fas1-4 rad51-3*	*fas1-4 rad51-2*
WT		***	***	***	***	***
fas1-4			NS	NS	NS	*
fas2-1				NS	NS	*
fas1-4 dmc1-2					NS	*
fas1-4 rad51-3						NS
fas1-4 rad51-2						

Asterisks indicate *P*-values from chi-square test: NS, no significant; $^{***}P<0.001$ and $^{*}P<0.05$.

Supplementary Table S4. Statistical comparisons of the number of cells with dicentric chromosomes between the mutants analyzed.

Metaphase	WT	*fas1-4*	*fas2-4*	*fas1-4 dmc1-2*	*fas1-4 rad51-3*	*fas1-4 rad51-2*
WT		***	***	***	***	***
fas1-4			NS	NS	NS	NS
fas2-1				NS	NS	NS
fas1-4 dmc1-2					NS	NS
fas1-4 rad51-3						NS
fas1-4 rad51-2						

Asterisks indicate *P*-values from chi-square test: NS, no significant; ***P<0.001.

Supplementary Table S5. Statistical comparisons of the number of cells with more than three chromosomes fused between the mutants analyzed.

Metaphase	WT	*fas1-4*	*fas2-4*	*fas1-4 dmc1-2*	*fas1-4 rad51-3*	*fas1-4 rad51-2*
WT		NS	NS	NS	***	***
fas1-4			NS	NS	*	*
fas2-1				NS	*	*
fas1-4 dmc1-2					*	*
fas1-4 rad51-3						NS
fas1-4 rad51-2						

Asterisks indicate *P*-values from chi-square test: NS, no significant; $^{***}P<0.001$ and $^{*}P<0.05$.

Supplementary Table S6. Statistical comparisons of the number of cells with anaphase alterations between the mutants analyzed.

Metaphase	WT	*fas1-4*	*fas2-4*	*fas1-4 dmc1-2*	*fas1-4 rad51-3*	*fas1-4 rad51-2*
WT		***	**	**	***	***
fas1-4			NS	NS	*	**
fas2-1				NS	*	**
fas1-4 dmc1-2					*	**
fas1-4 rad51-3						NS
fas1-4 rad51-2						

Asterisks indicate *P*-values from chi-square test: NS, no significant; ****P*<0.001, ***P*< 0.01 and **P*<0.05.
